# Trends in Oxidative Balance Score and Prevalence of Metabolic Dysfunction-Associated Steatotic Liver Disease in the United States: National Health and Nutrition Examination Survey 2001 to 2018

**DOI:** 10.3390/nu15234931

**Published:** 2023-11-27

**Authors:** Zongbiao Tan, Yanrui Wu, Yang Meng, Chuan Liu, Beiying Deng, Junhai Zhen, Weiguo Dong

**Affiliations:** 1 Department of Gastroenterology, Renmin Hospital of Wuhan University, 238 Jiefang Road, Wuhan 430060, China; 2 Department of Ophthalmology, Renmin Hospital of Wuhan University, 238 Jiefang Road, Wuhan 430060, China; 3 Department of General Practice, Renmin Hospital of Wuhan University, 238 Jiefang Road, Wuhan 430060, China

**Keywords:** metabolic dysfunction-associated steatotic liver disease, prevalence, oxidative stress score, inflammatory status, insulin resistance

## Abstract

Background: Most studies have primarily focused on assessing the association between diet or exercise patterns and metabolic dysfunction-associated steatotic liver disease (MASLD). This study adopted a more comprehensive approach by introducing the oxidative balance score (OBS) to evaluate the combined effects of diet and lifestyle on the body’s antioxidant ability. Our main objective was to investigate the association between OBS and the burden of MASLD in the United States. Methods: Participants with complete information from 2001 to 2018 were included. In the absence of other definite liver injury factors, the United States fatty liver index (us-FLI) ≥ 30 was used as the diagnostic criterion for MASLD. We first calculated the weighted prevalence for each cycle and stratified it according to demographic and metabolic-related disease characteristics. Subsequently, weighted multiple logistic regression was used to evaluate the relationship between OBS and MASLD. In addition, we explored the body’s inflammatory state and the level of insulin resistance (IR) in mediating OBS and MASLD. Results: From 2001 to 2018, the prevalence of MASLD in the U.S. population as a whole increased from 29.76% to 36.04%, and the rate was higher in people with metabolic-related diseases. Notably, OBS exhibited a negative correlation with MASLD. Participants in the highest tertile of OBS had a significantly lower prevalence of MASLD compared to those in the lowest tertile [OR: 0.72, 95%CI: (0.57, 0.92), *p* < 0.001]. Moreover, a high OBS is associated with a lower inflammatory state and level of IR. The body’s inflammatory state and IR level mediated the association between OBS and MASLD by 5.2% and 39.7%, respectively (both *p* < 0.001). Conclusions: In this study, we observed an increasing prevalence of MASLD over the years. A higher OBS was associated with a lower risk of MASLD, especially when OBS ≥ 25. The body’s inflammatory state and IR level mediate the association between OBS and MASLD, but the mechanism needs to be further investigated.

## 1. Introduction

Metabolic-dysfunction-associated steatotic liver disease (MASLD), formerly known as non-alcoholic fatty liver disease (NAFLD), is a clinicopathological syndrome predominantly characterized by diffuse hepatocellular steatosis and fat due to causes other than alcohol and other definite liver injuries [1]. MASLD has become one of the world’s most common chronic liver diseases and public health problems [2]. In recent years, MASLD has been more and more commonly seen, mainly attributed to the prevalence of obesity and metabolic disorders. Approximately 38% of the global population is affected by MASLD, with a higher prevalence observed among individuals with obesity [3,4,5].

The pathogenesis of MASLD is very intricate. With the deepening of research, the classical “Two-hit” theory has been gradually replaced by the more comprehensive “Multiple-hit” hypotheses. The occurrence and development of MASLD are thought to involve a variety of factors, including genetic susceptibility, oxidative stress, insulin resistance (IR), mitochondrial dysfunction, and so on [1,6]. Despite significant advancements in understanding the underlying mechanisms of MASLD, there is still a lack of effective pharmaceuticals to intervene and treat MASLD. Given the role of oxidative stress in the pathogenesis of MASLD, a growing number of studies have focused on how to enhance the body’s antioxidant capacity to prevent or stop the progression of MASLD [7]. Available options include increasing dietary antioxidant intake and exercise. For example, an increased intake of antioxidant bioactive compounds, such as omega-3 polyunsaturated fatty acids and selenium, has been found to be associated with a lower risk of MASLD and liver steatosis [8,9]. Exercise can not only reduce weight but also enhance the body’s ability to resist oxidative stress, thus effectively reducing the risk of MASLD [10]. However, the above studies focused on the singular assessment of the relationship between diet or exercise and MASLD and ignored the impact of overall behavior (including diet and lifestyle) on MASLD. Compared with these studies, the oxidative balance score (OBS) comprehensively considered the effects of both diet and lifestyle on oxidative stress as well as the role of antioxidants in the body; therefore, it could more accurately reflect the overall situation [11]. The higher the OBS, the stronger the body’s antioxidant capacity. OBS has been widely used to assess the relationship with other chronic diseases, including chronic kidney disease and depression [12,13]. However, there is currently little evidence to assess the association between OBS and MASLD. A case–control study comparing OBS in MASLD patients with non-MASLD participants showed that the latter had higher OBS [14]. Another large Korean cohort observed that high levels of OBS were inversely associated with the incidence of MASLD after a long follow-up period [15]. However, whether the burden of MASLD in the United States population is related to OBS remains unknown. In addition, they did not assess whether the protective effect of OBS against MASLD was related to improved IR and reduced inflammatory states.

Therefore, this study used data from the National Health and Nutrition Examination Survey (NHANES) database to assess the burden of MASLD in the United States. Subsequently, we explored the impact of OBS on the burden of MASLD in the U.S. Furthermore, we used mediation analysis to investigate if IR and inflammatory states have mediated the relationship between OBS and MASLD.

## 2. Materials and Methods

The NHANES is a critical cross-sectional survey program conducted by the National Center for Health Statistics (NCHS) in the United States. The primary purpose of NHANES is to monitor nutrition and health-related issues nationwide. Utilizing a stratified, multi-stage sampling design, NHANES ensures the selection of a representative sample from the general population, enabling researchers to derive valuable insights into various health conditions, including MASLD.

### 2.1. Study Design and Population

**Figure 1:**
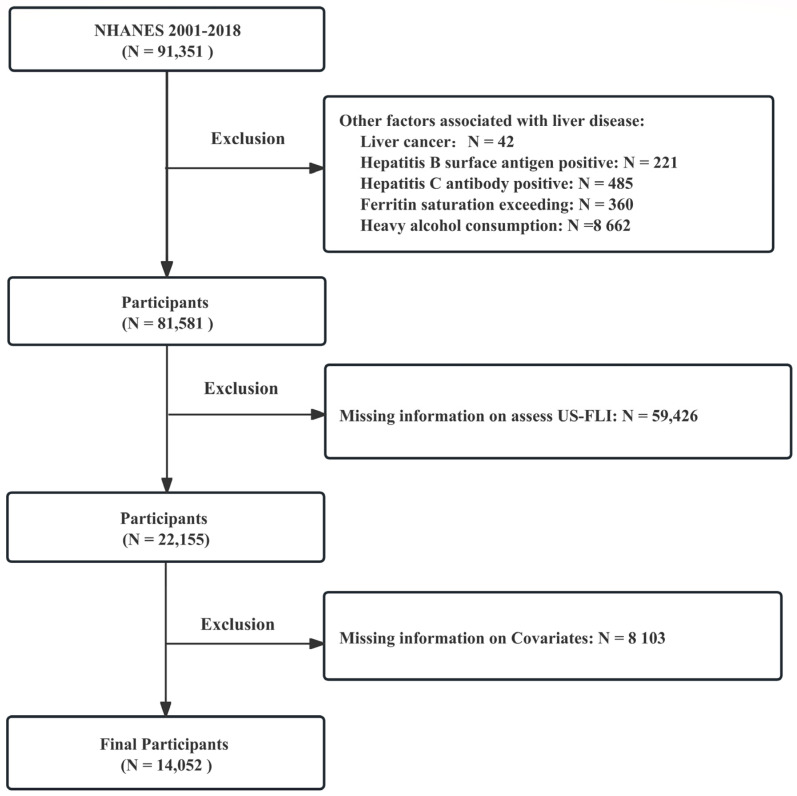
Flow chart of sample selection from the NHANES 2001–2018.

Data were extracted from NHANES, encompassing nine cycles from 2001 to 2018. The exclusion criteria used in this study were as follows: (1) participants with liver disease associated with other factors, including ① alcohol-related liver disease, characterized by heavy drinking (≥3 drinks per day for females and ≥4 drinks per day for males) or binge drinking (≥5 drinks on a single occasion); ② hepatitis virus infection, identified by the presence of hepatitis B surface antigen or hepatitis C confirmation antibody; ③ iron metabolic disorders, indicated by ferritin saturation exceeding 50%; and ④ self-reported liver cancer, (2) participants lacking information required to assess MASLD, and (3) participants with missing information on other relevant variables. The detailed flow is shown in Figure 1. According to the above criteria, the study population comprised 14,052 participants with complete information, including 10,391 non-MASLD patients (73.95%) and 3661 MASLD patients (26.05%).

### 2.2. Definition of MASLD

In addition to excluding other definite liver injury factors, the diagnosis of MASLD is usually based on abdominal ultrasonography, magnetic resonance imaging, and other imaging tests to detect liver fat, and further liver biopsy is required if necessary. Due to the high operator requirements, cost issues, and the fact that steatosis is only detected when more than 20–30% of liver cells have steatosis, it is not widely used. Therefore, CE Ruhl developed a score for assessing fatty liver disease in the U.S. population [16]. The United States fatty liver index (US-FLI) has good sensitivity and specificity and has been validated in other studies [17]. Therefore, this study mainly used us-FLI ≥ 30 as a criterion to diagnose MASLD.

### 2.3. Evaluation of Oxidative Balance Score (OBS)

The OBS encompasses an assessment of dietary and lifestyle factors, including dietary fiber, carotene (alpha- and beta-carotene), riboflavin, niacin, vitamin B6, total folate, vitamin B12, vitamin C, vitamin E, calcium, magnesium, zinc, copper, selenium, total fat intake, iron intake, body mass index (BMI), physical activity, and cotinine’s contribution to oxidative stress [11]. In the evaluation process, higher dietary antioxidant intake and more physical activities lead to increased OBS, indicating a more favorable antioxidant capacity. Conversely, higher intake of total fat, iron, cotinine, and elevated BMI result in lower OBS, reflecting increased levels of oxidative stress. The final OBS is derived by summing the assigned scores for each component. The specific distribution criteria of OBS components are in Supplementary Table S1.

### 2.4. Evaluation of the Body’s Inflammatory State

The Systemic Inflammatory Index (SII) was utilized as a metric to assess the body’s inflammatory state in this study. This indicator was originally used in the field of oncology to evaluate the inflammatory status and prognosis of patients, and as research continued to deepen, SII was found to be associated with liver steatosis [18]. Higher SII values generally correlate with elevated levels of systemic inflammation within the body. To compute the SII value, the following formula was employed: SII = P × N/L. (“P” represents the neutrophil count, “N” corresponds to the lymphocyte count, and “L” denotes the platelet count) [19]. 

### 2.5. Evaluation of the Body’s Insulin Resistance (IR) Level

Insulin sensitivity was assessed using the homeostasis model of insulin resistance (HOMA-IR). A higher HOMA-IR value indicates reduced sensitivity to insulin, suggesting the presence of IR [20]. The specific calculation formula for HOMA-IR is as follows: HOMA-IR = (fasting insulin × fasting blood glucose)/22.5.

### 2.6. Covariates

In this study, we considered several covariates which could potentially confound the outcomes. These variables encompassed demographic characteristics and risk factors associated with metabolism-related diseases. The demographic characteristics included (1) age; (2) gender (male and female); (3) race (Mexican American, White, Black, and other races); and (4) education level (below high school, high school, and more than high school). As for the risk factors related to metabolism, we accounted for hypertension (yes/no), diabetes (yes/no), and hyperlipidemia (yes/no). Additionally, BMI and total energy intake were also adjusted for in the analysis to ensure their influence on the outcomes was appropriately considered.

### 2.7. Statistical Analysis

To ensure the data’s representativeness for the entire U.S. population, we applied the recommended weights by the NCHS. Firstly, we calculated the weighted prevalence and 95% confidence intervals (CI) for MASLD from 2001 to 2018. To identify the prevalence of MASLD within specific subgroups defined by gender, race, BMI, diabetes, hypertension, and hyperlipidemia, we stratified the overall population and calculated the prevalence within these subgroups. Then, descriptive statistics were used to describe participants’ characteristics. The categorical variables used chi-square tests (presented as percentages), while continuous variables were compared using *t*-tests (presented as mean ± standard deviation). To explore the relationship between OBS and MASLD. Weighted multiple linear regression and weighted multiple logistic regression were used to analyze the effect relationship of OBS on US-FLI and the association between OBS and MASLD, respectively. In this process, we gradually adjust the covariates to build three models. Model 1 adjusted for demographic characteristics, including age, sex, ethnicity, and education level. Model 2, based on Model 1, further adjusted for total energy intake and liver enzymes (alanine transaminase [ALT], aspartate transaminase [AST]). Model 3 extended Model 2 by incorporating metabolism-related factors (BMI, hypertension, hyperlipidemia, and diabetes). To explore potential nonlinear relationships and threshold effects between OBS, US-FLI, and MASLD, we utilized restricted cubic spline (RCS). Furthermore, we conducted a mediation analysis to investigate if SII and HOMA-IR have mediated OBS in MASLD. All mediation models were adjusted for age, sex, ethnicity, education, total energy intake, liver enzymes (ALT, AST), BMI, hypertension, hyperlipidemia, and diabetes.

## 3. Results

### 3.1. Prevalence of MASLD in the U.S. Population from 2001 to 2018

Table 1 presents the overall prevalence of MASLD in the United States general population during the period from 2001 to 2018, along with the prevalence in different subgroups. Over this time, the prevalence of MASLD in the U.S. general population showed an upward trend, increasing from 26.21% to 36.04%. Further analysis by gender revealed that the prevalence of MASLD was lower in women compared to men; however, the rate of increase in women was notably higher than that in men. Regarding ethnic groups, Mexican Americans exhibited the highest prevalence of MASLD, while Black people had the lowest prevalence. Compared with the general population, the prevalence of MASLD is higher in patients with pre-existing obesity, hypertension, hyperlipidemia, and diabetes. Of note, in people with diabetes, the prevalence rate is as high as 70% or even higher.

### 3.2. Baseline Characteristics of the Study Population

Table 2 presents a comprehensive overview of the survey-weighted characteristics of the study population, categorized according to the presence or absence of MASLD. A total of 14,052 participants were included in the study, and the mean age of all participants was 43.59 years, with 48.97% being women. Among the total participants, MASLD was observed in 3661 individuals (26.05%) and was more prevalent in males than females (59.16% vs. 40.84%). Compared with non-MASLD participants, MASLD participants were generally older and had a higher proportion of education. Meanwhile, MASLD patients exhibited higher energy intake, inflammation, IR, and liver enzyme levels. In addition, MASLD patients have higher rates of hypertension, diabetes, and hyperlipidemia. The OBS levels were notably lower in individuals with MASLD (20.32) in comparison to those without MASLD (21.76).

### 3.3. Relationship between OBS and US-FLI and MASLD

**Figure 2:**
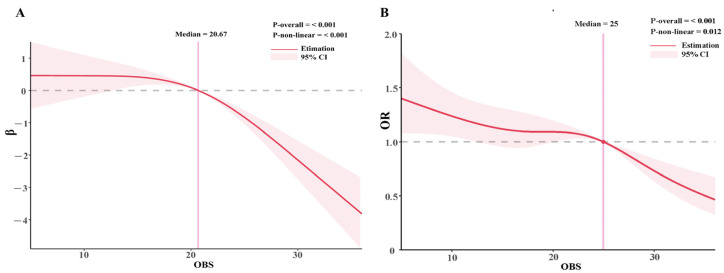
Relationship between OBS and US-FLI and MASLD. (**A**) Restricted cubic spline analysis of OBS for the estimation of US-FLI; (**B**) restricted cubic spline analysis of OBS for the estimation of the risk of MASLD. Restricted cubic spline model adjusted for age, sex, ethnicity, education, total energy intake, liver enzymes (ALT, AST), BMI, hypertension, hyperlipidemia, and diabetes. (MASLD = Metabolic dysfunction-associated steatotic liver disease; OBS =oxidative balance score; US-FLI = United States fatty liver index; BMI = body mass index; OR= odds ratio).

The weighted multiple linear regression analysis revealed a significant and consistent negative correlation between the OBS and the US-FLI (Table 3). In Model 1, which included adjustments for demographic characteristics, the observed results were as follows: [β: −0.43, 95% CI: (−0.49, −0.36), *p* < 0.001]. Model 2 continued to demonstrate a strong negative relationship between OBS and US-FLI: [β: −0.65, 95% CI: (−0.73, −0.57), *p* < 0.001]. Even after full adjustments in Model 3, the negative correlation persisted: [β: −0.17, 95% CI: (−0.22, −0.12), *p* < 0.001]. The relationship between OBS and MASLD also followed a similar pattern (Table 3). In Model 1 and Model 2, participants with Q2 and Q3 levels of OBS were negatively associated with the occurrence of MASLD when compared with participants in the first tertile (Q1) (*p* < 0.05). However, in Model 3, only the participants with the highest level (Q3) of OBS exhibited a similar protective effect on MASLD [Model 3: OR: 0.72, 95% CI: (0.57, 0.92), *p* < 0.001]. The relationship between OBS and US-FLI or MASLD was further examined using RCS, which revealed a non-linear association described as an inverted “L” shape. Liver steatosis significantly decreased when OBS > 20.67, and the risk of MASLD was notably reduced when OBS ≥ 25 (Figure 2A,B).

### 3.4. The Mediation Analysis between OBS and MASLD

**Figure 3:**
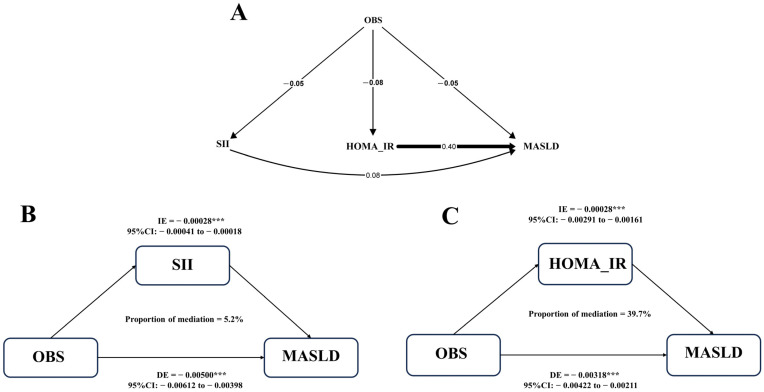
Mediation analysis between OBS and MASLD. (**A**) Pathway models show associations between HOMA-IR, SII, and OBS with MASLD; (**B**) estimated proportion of the association between OBS and MASLD mediated by SII; (**C**) estimated proportion of the association between OBS and MASLD mediated by HOMA-IR. Model adjusted for age, sex, ethnicity, education, total energy intake, liver enzymes (ALT, AST), BMI, hypertension, hyperlipidemia, and diabetes. (MASLD = Metabolic dysfunction-associated steatotic liver disease; OBS = oxidative balance score; BMI = body mass index; SII = Systemic Inflammatory Index; HOMA-IR = Homeostasis model of insulin resistance; CI = Confidence interval; DE = Direct effect; IE = Indirect effect, ***: p < 0.001).

The body’s inflammatory status and IR are important factors leading to MASLD. The results of the pathway model were consistent as expected (Figure 3A). SII and HOMA-IR were significantly positively correlated with MASLD, with effects of 0.08 and 0.40, respectively (Both *p* < 0.05). Results of mediation analysis showed that the proportions of SII and HOMA-IR mediating OBS and MASLD were 5.2% and 39.7%, respectively (Figure 3B,C). OBS is negatively correlated with SII and HOMA-IR (both *p* < 0.05), indicating that higher OBS may be associated with a lower level of the body’s inflammatory state and IR.

## 4. Discussion

In this nationally representative cross-sectional study, we investigated the prevalence of MASLD in the U.S. population over the period from 2001 to 2018. Our findings revealed a concerning increase in the overall prevalence of MASLD from 26.21% to 36.04% during this time frame. Notably, individuals with metabolism-related diseases exhibited higher rates of MASLD, underscoring the significance of these conditions as risk factors. On a positive note, our study highlighted a promising finding concerning the relationship between OBS and MASLD. Higher OBS levels demonstrated a protective effect on developing MASLD, particularly when OBS ≥ 25. This suggests that improving the body’s antioxidant capacity via dietary and lifestyle interventions may be beneficial in reducing the risk of MASLD. To delve deeper into this association, we conducted a mediation analysis, which shed light on the underlying mechanisms. The results of the mediation analysis indicated that increasing OBS may be associated with a lower body’s inflammatory state and IR levels. 

Based on a 2016 meta-analysis, the estimated prevalence of MASLD in North America was around 24% [21]. Due to the influence of obesity and metabolic diseases, a new study in 2023 shows that the prevalence of MASLD in North America has increased to 31.2% [5]. These findings are consistent with our study, which also revealed a significant increase in the prevalence of MASLD. Moreover, our results indicate that this rising trend is evident across all ethnic groups, women, individuals with obesity, non-hypertensive individuals, and subgroups with hyperlipidemia. This signifies the urgency of strengthening the management and intervention strategies for MASLD patients and high-risk individuals [22,23]. However, there is currently a lack of effective drugs to treat MASLD and exercise and diet modification are still the mainstream ways to prevent and treat MASLD. Numerous studies have consistently demonstrated a negative correlation between physical activity and MASLD. Importantly, this association extends beyond individuals engaged in rigorous physical activity to encompass individuals maintaining light and moderate levels of exercise. In other words, even light to moderate physical activity yields multiple benefits in the prevention and treatment of MASLD [10,24]. Regarding dietary habits, poor eating patterns can elevate the risk of obesity and IR, thereby promoting the development of MASLD. In contrast, adopting high-quality dietary patterns, such as the Mediterranean diet, dietary approaches to stop hypertension diet, and a very low-calorie ketogenic diet has been found to exert a protective effect against MASLD [25,26,27]. Among various dietary patterns, the Mediterranean diet has gained widespread recognition for its beneficial effects in reducing the risk of metabolic syndrome, diabetes, and coronary heart disease [23,28]. Unlike other diets, the Mediterranean diet emphasizes a predominantly plant-based approach and incorporates olive oil, a rich source of unsaturated fatty acids, as the primary fat source. A long-term clinical intervention trial conducted in Israel demonstrated that the green Mediterranean diet led to a greater reduction in liver fat infiltration compared to other healthy diets. This substantial reduction in liver fat infiltration is associated with a significant decrease in the risk of developing MASLD [29]. In our research, we aimed to assess the dose–response relationship between OBS and MASLD by considering OBS as an overall indicator of dietary and lifestyle modifications. Our findings were consistent with two previous studies [14,15], indicating a reduced risk of MASLD in individuals with higher OBS levels, especially when OBS ≥ 25.

The results of RCS show that the relationship between OBS and MASLD is not linear. Individuals with OBS ≥ 20.67 and OBS ≥ 25 have a lower risk of liver steatosis and MASLD, respectively, when compared to the general population. This is because OBS reflects the combined effect of prooxidant and antioxidants in the body, and the protective effect on MASLD will only show up after the antioxidant capacity reaches a certain level threshold. When OBS levels are low, oxidative stress predominates in the body. Oxidative stress refers to an imbalance between prooxidant and anti-oxidation processes in the body, and it has been closely linked to aging and the development of various diseases, including MASLD [30,31,32]. The Broad Institute identified oxidative stress as the cause of IR [33]. Oxidative stress triggers the buildup of reactive oxygen species (ROS) within the body. ROS activates pathways such as IKK/NF-KB, JNK/SAPK, and P13-K, which disrupt cellular insulin receptor signal transduction and downstream signaling pathways involving protein kinase B, lnsR, and IRS phosphorylation, further affecting the expression of glucose transporters [33,34]. Both oxidative stress and insulin resistance are important factors involved in the development of MASLD [1]. Conversely, when the body’s antioxidant capacity is higher than the level of oxidative stress, a protective effect on MASLD is shown. This is similar to the findings of previous studies, which showed that higher antioxidant intake and physical activity were associated with a lower risk of MASLD [10,35]. 

In this study, we observed a negative correlation between OBS and indicators representing the body’s inflammatory state and the level of IR. Both dietary antioxidants and exercise have the potential to elevate the activity of essential antioxidant enzymes like glutathione peroxidase (GPx), catalase (CAT), and superoxide dismutase (SOD) within the body. It could synergistically complement the endogenous free radical scavenging system, thereby effectively regulating the body’s antioxidant capacity [36,37]. Furthermore, exercise yields additional benefits. On one hand, it positively impacts the body’s insulin sensitivity, leading to a reduction in the influx of free fatty acids to the liver and lowering the levels of fat synthesis substrates [38]. On the other hand, exercise also influences the activity of fat synthase, effectively inhibiting the synthesis of liver fat [39]. Consequently, this dual effect assists in tuning the level of liver fat metabolism. Therefore, combining dietary antioxidants with physical activity can promote overall health.

Our study added new evidence emphasizing the importance of adopting an antioxidant-rich diet and lifestyle for managing individuals with high risk for MASLD. Given the rising prevalence of MASLD and its association with metabolism-related diseases, our findings underscore the potential role of antioxidants in MASLD prevention and management strategies. Promoting antioxidant intake and lifestyle modifications can contribute to reducing the burden of MAFLD and improving public health outcomes. Nevertheless, it is essential to acknowledge that this study did have some limitations. First, because this study was a retrospective cross-sectional survey, we cannot infer a causation link. Second, due to the inherent limitations of the NHANE study, the diagnosis of MASLD in this study was based on US-FLI. Other diagnostic methods, such as ultrasound and magnetic resonance imaging, are better at diagnosing MASLD and can provide a more intuitive assessment of fibrosis, but these methods are time- and money-consuming for large cohort studies. Novel, simpler, and more accurate diagnostic techniques will help to solve this problem. Third, although mediation analysis reflects that OBS may affect the occurrence of MASLD by influencing the inflammatory state and insulin resistance level of the body, more laboratory and clinical studies are still needed to explore the mechanism in the future.

## 5. Conclusions

Over the past years, the prevalence of MASLD in the United States has increased significantly from 29.76% to 36.04%, and this proportion is higher in people with metabolic-related diseases. However, we observed that higher OBS was associated with lower MASLD occurrence. The body’s inflammatory state and IR level may be involved in the association between OBS and MASLD, but the mechanism needs to be further explored.

## Figures and Tables

**Table 1:** The trend analysis of the weighted prevalence of MASLD in the U.S. population from 2001 to 2018.

Characters	2001–2002 (n = 1854)	2003–2004 (n = 1737)	2005–2006 (n = 1647)	2007–2008 (n = 1520)	2009–2010 (n = 1701)	2011–2012 (n = 1496)	2013–2014 (n = 1564)	2015–2016 (n = 1398)	2017–2018 (n = 1135)	*p*-Trend
Total	26.21 (23.14, 29.29)	23.75 (19.84, 27.66)	26.88 (23.30, 30.47)	27.33 (23.94, 30.72)	25.93 (23.22, 28.64)	26.24 (22.35, 30.13)	24.70 (22.23, 27.17)	29.53 (26.57, 32.49)	36.04 (32.13, 39.96)	<0.001
Sex										
Male	31.86 (26.20, 37.52)	31.17 (27.85, 34.50)	33.48 (29.39, 37.57)	33.76 (29.70, 37.82)	32.79 (28.97, 36.61)	28.52 (23.74, 33.30)	26.19 (22.10, 30.29)	35.55 (29.95, 41.15)	40.48 (33.77, 47.20)	0.211
Female	20.39 (16.05, 24.72)	16.18 (10.82, 21.54)	19.92 (15.37, 24.47)	20.90 (16.37, 25.43)	19.17 (15.45, 22.89)	24.15 (18.33, 29.98)	23.29 (20.30, 26.27)	23.78 (20.28, 27.27)	31.79 (25.62, 37.95)	<0.001
Race										
Mexican	34.28 (28.15, 40.41)	28.31 (19.94, 36.68)	34.37 (29.45, 39.28)	43.42 (38.03, 48.81)	39.38 (33.22, 45.53)	41.00 (33.12, 48.87)	34.90 (27.39, 42.41)	42.30 (33.44, 51.17)	50.56 (41.80, 59.32)	0.001
White	27.26 (23.84, 30.67)	25.03 (20.31, 29.75)	28.85 (24.41, 33.30)	27.89 (24.06, 31.73)	26.33 (22.95, 29.72)	26.18 (20.70, 31.66)	25.66 (22.17, 29.15)	29.89 (26.05, 33.74)	38.03 (33.03, 43.03)	0.006
Black	16.71 (13.00, 20.42)	13.48 (9.82, 17.14)	13.77 (10.44, 17.11)	13.19 (9.15, 17.23)	16.49 (10.67, 22.31)	17.81 (12.61, 23.00)	16.45 (11.40, 21.51)	18.43 (14.33, 22.53)	20.61 (15.98, 25.24)	0.013
Other	21.66 (14.27, 29.06)	21.20 (11.49, 30.90)	21.44 (11.46, 31.42)	27.49 (22.86, 32.11)	21.57 (16.09, 27.05)	24.43 (19.10, 29.76)	19.63 (15.72, 23.54)	30.22 (25.69, 34.76)	31.01 (23.65, 38.37)	0.042
BMI										
<25	3.67 (2.02, 5.31)	2.73 (1.06, 4.39)	2.11 (0.91, 3.31)	2.64 (0.95,4.33)	2.73 (1.38, 4.07)	1.86 (0.42, 3.31)	1.89 (0.31, 3.46)	3.07 (1.15, 4.98)	5.56 (2.55, 8.56)	0.590
≥25	42.65 (38.94, 46.37)	36.15 (31.88, 40.42)	41.90 (36.37, 47.43)	42.48 (37.27, 47.68)	43.27 (39.41, 47.13)	40.71 (35.23, 46.20)	38.62 (34.79, 42.44)	42.06 (38.88, 45.24)	48.80 (43.52, 54.09)	0.041
Diabetes mellitus										
No	22.08 (19.12, 25.04)	19.99 (16.11, 23.86)	22.09 (18.34, 25.85)	22.17 (18.21, 26.13)	21.57 (18.84, 24.30)	21. 29 (17.21, 25.37)	19.65 (17.07, 22.23)	22.80 (19.11, 26.48)	28.23 (23.78, 32.67)	0.081
Yes	73.74 (64.68, 82.80)	61.79 (54.69, 68.90)	66.71 (60.20, 73.22)	66.56 (57.92, 75.20)	61.33 (53.87, 68.78)	67. 85 (59.83,75.88)	64.20 (58.10, 70.30)	65.79 (58.18, 73.40)	78.76 (72.30, 85.21)	0.151
Hypertension										
No	17.27 (13.98, 20.56)	15.36 (13.14, 17.57)	17.66 (14.34, 20.99)	18.86 (15.62, 22.11)	17.22 (15.08, 19.36)	17.07 (13.09, 21.05)	17.31 (14.61, 20.01)	20.28 (16.15, 24.42)	24.88 (18.86, 30.89)	0.010
Yes	49.13 (39.56, 58.70)	40.99 (33.84, 48.15)	47.68 (42.21, 53.15)	46.29 (40.26, 52.32)	48.33 (42.58, 54.08)	48.65 (40.94, 56.36)	40.16 (36.48, 43.85)	46.55 (42.15, 50.95)	55.31 (48.10, 62.53)	0.261
Hyperlipidemia										
No	6.74 (4.07, 9.42)	10.27 (6.92, 13.62)	11.11 (6.95, 15.27)	7.83 (5.04, 10.62)	11.43 (8.19, 14.67)	6.58 (4.46, 8.70)	8.72 (5.95, 11.49)	12.26 (7.57, 16.94)	14.88 (8.91, 20.84)	0.056
Yes	34.98 (31.62, 38.35)	29.96 (25.53, 34.38)	34.76 (30.48, 39.04)	36.22 (32.21, 40.24)	33.92 (30.99, 36.85)	35.97 (30.84, 41.10)	33.75 (31.18, 36.32)	38.31 (33.87, 42.75)	46.48 (42.25, 50.71)	<0.001

Data were presented as weighted prevalence (%) and 95% CI. MASLD = Metabolic dysfunction-associated steatotic liver disease; BMI = body mass index, CI = confidence intervals.

**Table 2:** Baseline characteristics of the study population.

Variable	Total (n = 14,052)	Non-MASLD (n = 10,391)	MASLD (n = 3661)	*p* Value
**Age, Mean ± SD**	43.59 ± 0.29	41.00 ± 0.32	50.48 ± 0.34	<0.001
**US-FLI, Mean ± SD**	22.14 ± 0.27	11.18 ± 0.11	51.25 ± 0.37	<0.001
**BMI, Mean ± SD**	27.84 ± 0.09	25.54 ± 0.07	33.93 ± 0.14	<0.001
**Energy kcal, Mean ± SD**	2182.88 ± 11.88	2150.55 ± 12.79	2268.73 ± 23.37	<0.001
**ALT, Mean ± SD**	23.85 ± 0.26	20.78 ± 0.13	32.01 ± 0.88	<0.001
**AST, Mean ± SD**	24.22 ± 0.13	23.29 ± 0.15	26.68 ± 0.29	<0.001
**OBS, Mean ± SD**	21.37 ± 0.13	21.76 ± 0.14	20.32 ± 0.19	<0.001
**SII, Mean ± SD**	519.78 ± 3.76	503.37 ± 4.37	563.34 ± 6.52	<0.001
**HOMA_IR, Mean ± SD**	3.32 ± 0.05	1.97 ± 0.02	6.91 ± 0.16	<0.001
**BMI (%)**				<0.001
<25	5706 (40.61)	5536 (49.76)	170 (3.87)	
≥25	8346 (59.39)	4855 (50.24)	3491 (96.13)	
**Sex (%)**				<0.001
Female	6881 (48.97)	5324 (53.99)	1557 (40.84)	
Male	7171 (51.03)	5067 (46.01)	2104 (59.16)	
**Race (%)**				<0.001
Black	3117 (22.18)	2656 (11.74)	461 (6.08)	
Mexican	2490 (17.72)	1564 (5.95)	926 (10.04)	
Other	2361 (16.8)	1788 (11.72)	573 (10.22)	
White	6084 (43.3)	4383 (70.59)	1701 (73.66)	
**Educational level (%)**				<0.001
Below high school	2536 (18.05)	1986 (10.45)	550 (7.02)	
High school	5305 (37.75)	3892 (32.56)	1413 (35.47)	
More than high schools	6211 (44.2)	4513 (56.99)	1698 (57.51)	
**Hypertension (%)**				<0.001
No	9794 (69.7)	8037 (77.03)	1757 (45.88)	
Yes	4258 (30.3)	2354 (22.97)	1904 (54.12)	
**Diabetes mellitus (%)**				<0.001
No	12,218 (86.95)	9723 (94.88)	2495 (71.61)	
Yes	1834 (13.05)	668 (5.12)	1166 (28.39)	
**Hyperlipidemia (%)**				<0.001
No	5310 (37.79)	4792 (41.12)	518 (12.13)	
Yes	8742 (62.21)	5599 (58.88)	3143 (87.87)	

Continuous variables [presented as mean ± standard deviation], categorical variables [presented as percentages]. N = number; US-FLI = United States fatty liver index; BMI = body mass index; ALT = alanine transaminase, AST = aspartate transaminase; OBS = Oxidative Balance Score; SII = Systemic Inflammatory Index, HOMA-IR = homeostasis model of insulin resistance.

**Table 3:** Relationship between OBS and us-FLI and MASLD.

Outcome	Variable	Model 1	*p*	Model 2	*p*	Model 3	*p*
		β (95% CI)		β (95% CI)		β (95% CI)	
**Relationship between OBS and** **US-FLI**	OBS	−0.43 (−0.49, −0.36)	<0.001	−0.65 (−0.73, −0.57)	<0.001	−0.17 (−0.22, −0.12)	<0.001
		OR (95% CI)		OR (95% CI)		OR (95% CI)	
	OBS	0.96 (0.95, 0.97)	<0.001	0.94 (0.93, 0.95)	<0.001	0.98 (0.96, 0.99)	<0.001
**Relationship between OBS and MASLD**	Q1	ref	ref	ref	ref	ref	ref
	Q2	0.85 (0.74, 0.98)	0.027	0.72 (0.62, 0.85)	<0.001	0.88 (0.74, 1.06)	0.188
	Q3	0.60 (0.52, 0.70)	<0.001	0.46 (0.38, 0.55)	<0.001	0.72 (0.57, 0.92)	0.009

Model 1: adjusted for demographic characteristics, including age, sex, ethnicity, and education. Model 2: additionally adjusted for total energy intake and liver enzymes (ALT, AST) on the basis of Model 1. Model 3: additionally adjusted for metabolism-related factors (BMI, hypertension, hyperlipidemia, and diabetes) on the basis of Model 2. (MASLD = Metabolic dysfunction-associated steatotic liver disease; US-FLI = United States fatty liver index; BMI = body mass index; OR = odds ratio; CI = confidence intervals).

## Data Availability

The datasets for this study can be found in the National Health and Nutrition Examination Surveys database (https://www.cdc.gov/nchs/nhanes/index.htm, accessed on 13 August 2023).

## Supplementary Materials

The following supporting information can be downloaded at https://www.mdpi.com/article/10.3390/nu15234931/s1. Table S1. Components of the oxidative balance score (Zhang et al. (2022) [40]).
