# Supplementary material for: Trends in Oxidative Balance Score and Prevalence of Metabolic Dysfunction-Associated Steatotic Liver Disease in the United States: National Health and Nutrition Examination Survey 2001 to 2018
Source: Nutrients. 2023 Nov 27;15(23):4931. doi: 10.3390/nu15234931 (PMC10708172; doi:10.3390/nu15234931)
Supplement: Supplementary file 1 [file nutrients-15-04931-s001.zip › nutrients-2668297-supplementary.pdf]

nutrients

MDPI

Trends in Oxidative Balance Score and Prevalence of Metabolic Dysfunction-Associated Steatotic Liver Disease in the United States: National Health and Nutrition Examination Survey 2001 to 2018

Zongbiao Tan [1,†], Yanrui Wu [1,†], Yang Meng [2,†], Chuan Liu [1], Beiying Deng [1], Junhai Zhen [3] and Weiguo Dong [1,*]

1 Department of Gastroenterology, Renmin Hospital of Wuhan University, 238 Jiefang Road, Wuhan 430060, China
2 Department of Ophthalmology, Renmin Hospital of Wuhan University, 238 Jiefang Road, Wuhan 430060, China
3 Department of General Practice, Renmin Hospital of Wuhan University, 238 Jiefang Road, Wuhan 430060, China
* Correspondence: dongweiguo@whu.edu.cn; Tel.: +86-027-88041911
† These authors contributed equally to this work and share first authorship.

Table S1. Components of the oxidative balance score (Zhang et al. (2022) [1]).

OBS Components	Property	Male			Female		
		0	1	2	0	1	2
Dietary OBS components							
Dietary fiber (g/d)	A	<12.56	12.56-19.70	≥19.70	<10.10	10.10-16.31	≥16.31
Carotene (RE/d)	A	<98.83	98.83-306.25	≥306.25	<98.08	98.08-383.50	≥383.50
Riboflavin (mg/d)	A	<1.79	1.79-2.69	≥2.69	<1.34	1.34-2.02	≥2.02
Niacin (mg/d)	A	<20.65	20.65-29.75	≥29.75	<14.52	14.52-21.86	≥21.86
Vitamin B_6 (mg/d)	A	<1.59	1.59-2.40	≥2.40	<1.13	1.13-1.77	≥1.77
Total folate (mcg/d)	A	<316.00	316.00-492.00	≥492.00	<251.00	251.00-388.96	≥388.96
Vitamin B_{12} (mcg/d)	A	<3.36	3.36-6.20	≥6.20	<2.22	2.22-4.22	≥4.22
Vitamin C (mg/d)	A	<42.44	42.44-113.21	≥113.21	<38.01	38.01-98.49	≥98.49
Vitamin E (ATE) (mg/d)	A	<5.82	5.82-9.42	≥9.42	<4.53	4.53-7.52	≥7.52
Calcium (mg/d)	A	<646.00	646.00-1072.00	≥1072.00	<499.24	499.24-849.00	≥849.00
Magnesium (mg/d)	A	<257.00	257.00-361.28	≥361.28	<187.00	187.00-283.43	≥283.43
Zinc (mg/d)	A	<9.75	9.75-15.10	≥15.10	<6.73	6.73-10.75	≥10.75
Copper (mg/d)	A	<1.12	1.12-1.57	≥1.57	<0.85	0.85-1.28	≥1.28
Selenium (mcg/d)	A	<94.94	94.94-141.80	≥141.80	<67.79	67.79-99.50	≥99.50
Total fat (g/d)	P	≥69.83	69.83-107.43	<107.43	≥50.98	50.98-75.79	<75.79
Iron (mg/d)	P	≥12.88	12.88-19.17	<19.17	≥9.65	9.65-14.32	<14.32
Lifestyle OBS components							
Physical activity (MET-minute/week)	A	<417.86	417.86-1135.71	≥1135.71	<270.00	270.00-845.71	≥845.71
Alcohol (g/d)	P	≥30	0-30	None	≥15	0-15	None
Body mass index (kg/m^2)	P	≥25.54	25.54-29.17	<29.17	≥23.74	23.74-28.64	<28.64
Cotinine (ng/mL)	P	≥0.038	0.038-1.13	<1.13	≥0.035	0.035-0.172	<0.172

OBS: oxidative balance score; A: antioxidant; P: prooxidant; RE: retinol equivalent; ATE: alpha-tocopherol equivalent; MET: metabolic equivalent.

Reference

1. Zhang, W.; Peng, S.F.; Chen, L.; Chen, H.M.; Cheng, X.E.; Tang, Y.H. Association between the Oxidative Balance Score and Telomere Length from the National Health and Nutrition Examination Survey 1999–2002. *Oxidative Med. Cell. Longev.* **2022**, *2022*, 1345071. https://doi.org/10.1155/2022/1345071.

Disclaimer/Publisher's Note: The statements, opinions and data contained in all publications are solely those of the individual author(s) and contributor(s) and not of MDPI and/or the editor(s). MDPI and/or the editor(s) disclaim responsibility for any injury to people or property resulting from any ideas, methods, instructions or products referred to in the content.
